# Anxiety and depression among infertile women: a cross-sectional survey from Hungary

**DOI:** 10.1186/s12905-017-0410-2

**Published:** 2017-07-24

**Authors:** Enikő Lakatos, Judit F Szigeti, Péter P Ujma, Réka Sexty, Piroska Balog

**Affiliations:** 1 Institute of Behavioural Sciences, Semmelweis University, Budapest, Nagyvárad tér 4, Budapest, H-1089 Hungary; 2 Department of Clinical Psychology, Semmelweis University, Tömő utca 25-29, Budapest, H-1083 Hungary; 3 Institute of Medical Psychology, Centre for Psychosocial Medicine, Ruprecht-Karls University, Bergheimer Straße 20, D-69115 Heidelberg, Germany

**Keywords:** Infertility, Infertility-related distress, Depression, Anxiety

## Abstract

**Background:**

Infertility is often associated with a chronic state of stress which may manifest itself in anxiety-related and depressive symptoms. The aim of our study is to assess the psychological state of women with and without fertility problems, and to investigate the background factors of anxiety-related and depressive symptoms in women struggling with infertility.

**Methods:**

Our study was conducted with the participation of 225 (134 primary infertile and 91 fertile) women, recruited in a clinical setting and online. We used the following questionnaires: Spielberger Trait Anxiety Inventory (STAI-T), Shortened Beck Depression Inventory (BDI) and Fertility Problem Inventory (FPI). We also interviewed our subjects on the presence of other sources of stress (the quality of the relationship with their mother, financial and illness-related stress), and we described sociodemographic and fertility-specific characteristics. We tested our hypotheses using independent-samples t-tests (M ± SD) and multiple linear regression modelling (ß).

**Results:**

Infertile women were younger (33.30 ± 4.85 vs. 35.74 ± 5.73, *p* = .001), but had significantly worse psychological well-being (BDI = 14.94 ± 12.90 vs. 8.95 ± 10.49, *p* < .0001; STAI-*T* = 48.76 ± 10.96 vs. 41.18 ± 11.26, *p* < .0001) than fertile subjects. Depressive symptoms and anxiety in infertile women were associated with age, social concern, sexual concern and maternal relationship stress. Trait anxiety was also associated with financial stress. Our model was able to account for 58% of the variance of depressive symptoms and 62% of the variance of trait anxiety.

**Conclusions:**

Depressive and anxiety-related symptoms of infertile women are more prominent than those of fertile females. The measurement of these indicators and the mitigation of underlying distress by adequate psychosocial interventions should be encouraged.

## Background

### Psychosocial and mental aspects of women’s reproductive problems

Infertility is defined by the failure to achieve a clinical pregnancy after 12 months or more of regular unprotected sexual intercourse [1]. According to international estimates, the prevalence of infertility is about 9–15%, [2–4], where 9% refers to international prevalence of current infertility [2], while 10–15% to lifetime prevalence in Western societies [3]. Fisher [4] points out differences between point and lifetime prevalence data, and between definitions of infertility (primary, secondary etc.), which can also influence estimates. In Hungary, infertility occurs with a frequency similar to the international average [5].

Infertility is often associated with psychological strain, which can be both a cause and a consequence of the disorder [6]. The primary negative emotional response to both infertility and assisted reproductive treatment (ART) is usually either anxiety (a sense of threat, tension, worry) or depression (a sense of loss, sadness, lack of control) [7].

Based on an overview of 10 years of research, Greil et al. stated that infertile women are more likely to experience higher levels of distress than comparison groups [8]. Another review article confirms elevated depression levels in infertile women relative to fertile females [9]. Representative population-based studies found an increased likelihood of anxiety in subfecund women compared to fecund controls [10, 11].

Yet another systematic review of 25 years of research [12] found that, when assessed with standardized measures, women starting in vitro fertilization (IVF) were only slightly different emotionally from the norm groups. It has been argued that instruments for the measurement of general emotional adjustment are unlikely able to capture the specific distress associated with infertility and its treatment [13]. Therefore, it has become common to use questionnaires specifically developed to measure infertility-related distress [14].

Recently it has been reasoned that fertility treatment in itself may have negative psychological effects [13], possibly reducing its efficacy. However, most of what is known about infertility psychology is based on IVF patients; few studies have examined infertile women not receiving treatment.

The psychological response to assisted reproduction is mediated by both protective and risk factors [15]. Models of these relationships are typically circular: they consider the complex interactions between biological, psychological and social processes. Support from the social environment, especially from the partner, can be associated with decreased distress in infertile women [16, 17]. Conversely, infertility itself can also affect the quality of relationships. As a disorder of a couple as a functional unit, the inability to conceive (especially in the presence of inadequate communication) frequently leads to a lack of sexual satisfaction [18] or even a relationship crisis [19]. Other aspects of the social environment, e.g. social pressure (a perceived expectation of motherhood) can also worsen the psychological consequences of infertility [20]. The results concerning the stress-relieving effect of family support mainly come from studies viewing it as a global construct, without differentiating between various types of kinship, e.g. the quality of the infertile woman’s relationship to her mother.

Infertility is associated with a variety of other psychosocial and biological factors which may also affect its occurrence and severity. One of these factors is the age of the female which does not only influence the time between planning a pregnancy and successful conception, but also the healthy development and normal birth of the child [21]. Therefore, because of the increasing tendency to delay childbirth in Europe, advanced age can become a prominent psychosocial stress factor for infertile women.

Socioeconomic status may also affect fertility distress, but research results in this area are inconclusive. A probability-based study [13] supported the consistent finding that general distress levels are lower with higher socioeconomic status [22, 23], but found that fertility-specific distress is only related to age and no other demographic variable. Work-related stressors, such as concern about finances [16] or missing work [24], may also have an impact on psychological well-being, as well as reproductive outcome.

The goal of this study is to compare the mental well-being of fertile and infertile women in Hungary, and to identify some possible factors behind increased levels of depression and anxiety in infertile women.

## Methods

### Aims of the present study

We compared the mental status of fertile and infertile females with questionnaires either used in assessing the general population [Beck Depression, Inventory (BDI), Spielberger Trait Anxiety Inventory (STAI-T] or specially developed for infertile women [Fertility Problem Inventory, (FPI)]. To overcome some of the shortcomings listed in the above section, only primary infertile women who had never been pregnant before were included in our infertile sample. In order to identify possible factors behind increased levels of depression and anxiety, we assessed demographic and infertility-specific variables, financial difficulties, as well as stress rooted in the infertile woman’s relationship to her mother. Finally, since infertility is a medical diagnosis, the possible contribution of illness stress to mental status was also examined.

### Study design and data collection

Our cross-sectional study is based on clinical and online samples. Data were collected between September 2013 and September 2014 in two Budapest-based private fertility centers (Kaáli Institute and Forgács Institute) and in the publicly funded Obstetrics and Gynecology Clinic No 2 of Semmelweis University. The study questionnaires were also available online on the web page of the Institute of Behavioral Sciences, Semmelweis University (http://meddoseg.magtud.hu/) as well as on a Hungarian website dedicated to reproductive health (http:\\teherbeeses.hu). Participation in the study was voluntary and anonymous. Informed consent was obtained from all participants before data collection. The selection criteria for the study were fluent command of the Hungarian language, female gender and reproductive age (20–45). Subjects in the fertile group had to be either pregnant and/or the biological parent of at least one child. Subjects in the infertile group failed to conceive for at least 1 year despite their willingness and an active sexual life, and also had a lack of pregnancy in patient history (primary infertile). Secondary infertile women (having a previous live birth but recently struggling with fertility problems) were also identified during the data collection, but not included in the study sample.

A total of 225 women participated in our study, 134 of them with primary infertility (6 recruited in a clinical setting and 128 online) and 91 fertile controls (26 recruited in a clinical setting and 65 online). All women suffering from other chronic diseases (such as heart disease, autoimmune or hemorrhagic disease, diabetes, or hypertension) beyond infertility (*N* = 23) were excluded from the analysis.

The infertile group consisted of two groups. The first group included 103 women undergoing first time/successive ART treatment (insemination or IVF-cycles), and the second group included 31 women without a history of ART.

### Measurement tools

We used standardized and validated as well as study-specific questionnaires in order to assess certain psychological parameters related to reproductive health.

The level of trait anxiety was assessed with the Hungarian version [25] of the Spielberger State-Trait Anxiety Inventory [26]. We only used the Trait scale of the questionnaire (STAI-T), which refers to the general tendency of an individual to be anxious, and is assessed through Likert scale responses to questions such as “I feel that difficulties are piling up so that I cannot overcome them” or “I become tense and upset when I think about my present concerns”. A higher score indicates greater levels of anxiety. The Cronbach alpha value of this scale in our sample was .91. The score of 1 SD above the mean was used as the threshold for clinically relevant degrees of anxiety [27], this was 53 points (STAI-*T* < 45 no anxiety; 45–53 low or subclinical anxiety; >53 severe anxiety).

The prevalence of depressive symptoms was assessed with the shortened Hungarian version [28] of the Beck Depression Inventory (BDI) [29]. This questionnaire contains nine items with four-step (0–3) Likert scale responses about usual symptoms of depression such as pessimism, lack of satisfaction, guilt, social withdrawal, being indecisive, inhibition from work, sleep disturbances, fatigue, and somatic preoccupation. A higher score indicates a greater degree of depressed mood. Results on this scale can range from 0 to 27. We transformed the raw scores in order to make them comparable to the original (non-abbreviated) scale results which can assume values between 0 and 63. In our sample, this questionnaire yielded a Cronbach alpha score of .88. Based on the converted BDI scores, respondents were categorized in the following groups: normal (0–9), mild (10–18), moderate (19–25), and severe depression (≥26 points) [28].

Infertility-related distress was measured by the Hungarian version of the Fertility Problem Inventory (FPI) [14]. This questionnaire includes 46 items with six-step (1–6) Likert scale responses concerning the level of distress associated with the experience of infertility and parental roles. The subscales of this questionnaire are the following: Social Concern (e.g. “Family get-togethers are especially difficult for me”), Sexual Concern (e.g. “I find I’ve lost my enjoyment of sex because of the fertility problem”), Relationship Concern (e.g. “I can’t show my partner how I feel because it will make him/her feel upset”), Rejection of Childfree Lifestyle (e.g. “Couples without a child are just as happy as those without children” – reverse item) and Need for Parenthood (e.g. “Pregnancy and childbirth are the two most important events in a couple’s relationship”). A higher score indicates a more accentuated level of infertility-related distress. The original questionnaire was procured from the authors in English, and a Hungarian translation was created with their permission. The translation and re-translation was performed by the first author of this study and external collaborators, according to international guidelines. When comparing the two translations, we found their contents to be similar, which was also approved by the original authors. Cronbach alpha values for the subscales are (in order of the above appearance): .76, .81, .87, .80 and .74, respectively, while the full questionnaire yielded a Cronbach alpha value of .91.

Sources of stress were assessed with items using six-step (1–6) Likert scale responses. Prompted by the question “What sources of stress are present in your life?” the following responses were possible: “stress because my relationship is not harmonious 1) with my mother (hereafter, maternal relationship stress) 2) with my father 3) with my sibling(s) 4) with my partner 5) with my child(ren) 6) stress because of my illness (hereafter, illness stress) 7) stress rooted in my close relative’s illness 8) stress because of financial problems”. Only those variables which the professional literature has found relevant in coping with infertility were included in our study. Therefore, we mainly considered the partner [30] and the mother [31] as potential sources of interpersonal stress, while also including financial stress [32] and illness stress [33]. As one of the subscales of the Fertility Problem Inventory (Relationship Concern) measures the quality of the relationship with the partner, out of concerns of multicollinearity we rejected taking into account the separate results on partner-related stress. Therefore, we only considered maternal relationship stress, financial stress and illness stress as factors in the multivariate model. Higher values indicated an increased presence of stress from a given source.

Our test battery also included a record of sociodemographic parameters (age, place of residence, educational level, family income) as well as infertility-specific parameters (duration of infertility, participation in fertility treatments and the number of ART cycles (insemination or IVF-cycles).

### Statistical analyses

We first compared the level of depressive and anxiety-related symptoms in fertile vs. infertile women, and next, in infertile women with or without ART history. In both cases, we used independent-samples t-tests. Characteristics of the infertile and the control group were compared with chi-square tests (Table 1). We compared values of anxiety to mean values obtained on the Hungarian validation of the STAI-T inventory [25] and values of depression to mean values of the 20–45 years age group obtained with permission from the population-based data of the latest Hungarian Epidemiological Survey (‘Hungarostudy 2013’), a cross-sectional representative survey of the Hungarian adult population [34].

**Table 1:** Descriptive data: socio-demographic and psychosocial characteristics of study population

	Primary infertile (*N* = 134)	Fertile (*N* = 91)	Statistical test	*p*
Age (Mean, SD)	33.30 ± 4.85	35.74 ± 5.73	*t* = −3.44	*p* = .001
Age at first child birth (Mean, SD)		28.08 ± 5.03		
Educational level [*N* (%)]				
≤ 8 years	9 (6.72)	6 (6.60)	χ2 = 0.225	*p* = .849
9–13 years	40 (29.85)	24 (26.37)		
≥ 14 years	82 (61.19)	57 (62.64)		
Family income [*N* (%)]				
< 150.000 HUF^a^	72 (53.72)	72 (79.12)	χ2 = 16.181	*p* < .001
> 151.000 HUF	62 (46.26)	18 (19.78)		
Residence [*N* (%)]				
Village	24 (17.91)	15 (16.48)	χ2 = 10.529	*p* = .005
City	67 (50.00)	28 (30.77)		
Capital	43 (32.09)	48 (52.75)		
Duration of infertility (Mean, SD)	3.61 ± 3.08			
Numbers of ART (Mean, SD)	1.69 ± 2.64			
No participated in ART [*N* (%)]	31 (23.13)			
Depression^b^ (Mean, SD)	14.94 ± 12.90	8.95 ± 10.49	*t* = 3.68	<.001
Anxiety^c^ (Mean, SD)	48.76 ± 10.96	41.18 ± 11.26	*t* = 5.04	<.001

^a^150,000 HUF is approximately 500 EUR and is just about the average net income in Hungary

^b^BDI

^c^STAI-T

In order to identify underlying sources of depressive and anxiety-related symptoms in women with infertility problems, in the infertile group we implemented a linear regression analysis with depression and trait anxiety as dependent variables of two different models. Variebles were selected a priori based on the literature viewing the psychosocial background of female infertility globally (demographic variables: age [35], years of education [23]; infertility-specific variables: subscales of the Fertility Problem Inventory [17, 30]) and a specific construct (sources of stress: Financial Stress [32], Maternal Relationship Stress [31] and Illness Stress [33]). Thus, in both models, the independent variables were the following: 1. demographic variables: age, years of education (level of schooling transformed into number of years customarily spent in education); 2. infertility-specific variables: duration of infertility, number of fertility treatments (insemination or IVF-cycles); 3. subscales of the Fertility Problem Inventory (Social Concern, Sexual Concern, Relationship Concern, Rejection of Childfree Lifestyle, Need for Parenthood); 4. sources of stress: Financial Stress, Maternal Relationship Stress and Illness Stress. These regression analyses were performed on the infertile group only.

In our analysis, independent variables were all scale variables. Infertility-related differences in BDI and STAI-T scores persisted after correcting for demographic and other confounding variables: 14.940 (95% CI: 12.765–17.116, *p* < 0.001) and 48.761 (95% CI: 46.912–50.610, *p* < 0.001) before, and 14.610 (95% CI: 12.425–16.795, *p* < 0.001) and 48.546 (95% CI: 46.658–50.435, *p* < 0.001) after correction. All independent variables were added to the model without the application of stepwise or hierarchical modelling. All statistical analyses were performed with IBM SPSS 20.0 statistical software. The traditional significance threshold of *p* < .05 was used.

## Results

Concerning age, our results show that, at the time of data collection, the mean age (M ± SD) of the infertile population was younger than the fertile group (33.30 ± 4.85 vs. 35.74 ± 5.73, *p* = .001). However, one should note that, at the time of delivering their first child, fecund women were significantly younger than primary infertile women presently trying to conceive (28.08 ± 5.03 vs. 33.30 ± 4.85, *p* < .001). As far as residence is concerned, the proportion of those living in the capital was higher among fertile women, while town-dwellers were more numerous among infertile women. No difference was detectable in education (*p* = .849): both groups displayed higher levels of schooling. A larger income, however, was more frequent in the infertile group than in the fertile one (*p* < .001).

### Psychological well-being of fertile and infertile women

The mean values (M ± SD) of depressive symptoms (14.94 ± 12.90 vs. 8.95 ± 10.49, *p* < .001) and anxiety (48.76 ± 10.96 vs. 41.18 ± 11.26, *p* < .001) were significantly higher in infertile women when compared to fertile controls (Table 1). Involuntarily childless women were significantly more depressive (14.94 ± 12.90 vs. 5.44 ± 9.42 [34], *p* < .001) and more anxious (48.76 ± 10.96 vs. 45.37 ± 7.97 [36], *p* < .001) than the Hungarian female population of the same age group.

As for the prevalence of clinically significant depressive symptoms, 44.8% (95% CI: 36.4–53.1) of the infertile population (as opposed to 24.2% (95% CI: 15.7–32.9) of the fertile group) showed clinically relevant (moderate to severe) levels of depression. As far as anxiety is concerned, 39.6% (95% CI: 31.2–47.8) of the infertile group (as opposed to 17.6% (95% CI: 10.0–26.3) of the fertile sample) displayed high levels of trait-anxiety. Finally, 37.3% (95% CI: 28.7–45.3) of the infertile group showed a comorbidity of anxiety and depression (as opposed to 17.6%, (95% CI: 10.0–26.1) of the fertile group.

### Psychological well-being of infertile women with or without ART history

We found that ART patients had significantly more depressive symptoms than infertile women with no fertility treatments (15.74 ± 13.23 vs. 12.27 ± 11.54, *p* < .05). However, there was no significant difference between these groups in terms of trait anxiety (48.99 ± 10.71 vs. 48.00 ± 11.92, *p* = .350) and infertility-related stress (160.35 ± 33.89 vs. 156.00 ± 40.49, *p* = .196) (Table 2).

**Table 2:** Depressive and anxiety-related symptoms in infertile women with or without ART

[Mean, SD]	Infertile group with ART (*N* = 103)	Infertile group without ART (*N* = 31)	t value	*p*
Depression (BDI)	15.74 ± 13.23	12.27 ± 11.54	2.67	.009
Anxiety (STAI-T)	48.99 ± 10.71	48.00 ± 11.92	.94	.350
Fertility Problem Inventory (FPI)	160.35 ± 33.89	156.00 ± 40.49	1.30	.196

### Background factors of depressive and anxiety-related symptoms

In the infertile group we performed two linear regression analyses in order to identify predictors of depressive symptoms (BDI) and trait anxiety (STAI-T). We found that depressive symptoms were significantly associated with age (ß = .159, *p* < .018), Social Concern (FPI-1) (ß = .245, *p* < .003), Sexual Concern (FPI-2) (ß = .399, *p* < .001), and Maternal Relationship Stress (ß = .205, *p* < .002). The model explained 58% of the variance in depressive symptoms (R^2^ = .579, Adjusted R^2^ = .535, *p* < .001).

Similarly, trait anxiety was associated with age (ß = .142, *p* < .026), Social Concern (FPI-1) (ß = .315, *p* < .001), Sexual Concern (FPI-2) (ß = .303, *p* < .002), Financial Stress (ß = .173, *p* < .005) and Maternal Relationship Stress (ß = .162, *p* < .011), with a total explained variance of 62% (R^2^ = .615, Adjusted R^2^ = .575, *p* < .001) [Table 3].

**Table 3:** Predictors of depressive and anxiety-related symptoms

	Depression (BDI)^a^				Trait-anxiety(STAI-T)^a^			
Independent variables	Standardized beta	t value	*p*	R^2^/Adjusted R^2^	Standardized beta	t value	*p*	R^2^/Adjusted R^2^
Age^b^	.159	2.409	.018	.579/.535	.142	2.249	.026	.615/.575
Educational level^b^	.063	1.017	.311		−.030	−.508	.612	
Duration of infertility^b^	.066	1.009	.315		.054	.862	.391	
Number of assistant reproductive treatment^c^	.016	.235	.814		.026	.412	.681	
Social Concern	.245	3.072	.003		.315	4.125	.000	
Sexual Concern	.399	4.074	.000		.303	3.237	.002	
Relationship Concern	.038	.456	.649		.076	.948	.345	
Rejection of Childfree Lifestyle	.050	.609	.543		.137	1.745	.084	
Need For Parenthood	−.029	−.333	.740		.044	.527	.600	
Financial Stress	.065	1.026	.307		.173	2.837	.005	
Maternal Relationship Stress	.205	3.125	.002		.162	2.588	.011	
Illness stress	.113	1.735	.085		.012	.194	.847	

^a^Dependent variable

^b^years

^c^Number of procedures

## Discussion

Our study revealed significantly worse psychological status in infertile women in terms of both depression and trait anxiety compared with either a group of fertile women or the normative population. Almost half (44.8%) of our infertile sample displayed moderate to severe depressive symptoms. This result lends further support to previous Hungarian evidence of considerable depression (moderate to severe depression levels up to 27% [37] and 32% [38] among infertile women). More than a third (39.6%) of our infertile sample returned results indicative of clinical-level trait anxiety. The rate of infertile women with high trait-anxiety was 20% in an earlier Hungarian survey [38]. In addition, more than a third (37.3%) of our infertile sample, whereas less than a fifth of the fertile subjects (17.6%) displayed comorbid depression and trait anxiety. Our results are also in line with previous findings in other cultures reporting an increased level of trait anxiety [39, 40] and depressive symptoms [9, 41] associated with infertility.

As for the differences between infertile women undergoing assisted reproduction and those not, we found a higher level of depressive symptoms in ART patients. However, there was no difference in terms of either trait anxiety or infertility-related distress between the two groups. Previous review articles reported that (mostly unsuccessful) IVF treatments increased the probability of negative emotions [12], especially depression [9]. Repeated fertility treatments have sometimes been found to be even more predictive of infertility-related stress [13] and depression [42] than infertility itself. Our own results indicate that the effect of fertility treatments on psychological health may be the strongest in the case of depressive symptoms, possibly due to an increased sense of powerlessness [42].

In our study, infertile women were on average older than mothers at the time of their first delivery. Like in previous studies [35], older age was associated with higher level of depression in the infertile women. Compared with a few decades ago, in Europe (including Hungary) women tend to postpone childbearing, and advanced age is associated with impaired fertility [43, 44]. Depression could be the reaction to the potential shattering of a wish postponed until its fulfillment is doubtful.

Infertility-specific social concern was one of the strongest factors related to psychological status, and we assume that this association is possibly amplified by cultural factors, e.g. fertility expectations. Women’s motivations to have children include conformity to social norms and expectations [45], also depending on the original family background [46]. Social concern may express the individual’s reaction to social pressure for motherhood, which has been shown to be positively associated with infertility-related stress [20] and severe depressive symptoms [47]. Since the Hungarian society is one in which traditional values of mothering and child-orientedness still prevail [48], social concern about childlessness may well lead to higher levels of distress.

We found sexual concern to be another key variable connected to both trait anxiety and depression. Infertile women report more sexual problems, which is associated with an increased level of depressive symptoms [18]. Previous studies have shown that vegetative and subjective symptoms of general anxiety (nervousness, perspiration, abdominal discomfort) [19] or depression [37] are associated with more infertility-specific sexual concern. Thus, sexual concern might be a core manifestation of the severity of infertility-related psychological problems.

Interestingly, while partner support was elsewhere often found to be crucial in dealing with infertility-related psychological issues [30], relationship concern in our study did not correlate with psychological status. This suggests that loss of sexual self-esteem and of enjoyment, and feelings of pressure to schedule sexual intercourse are perceived by women as more disturbing than problems in communicating openly or constructively about infertility with the partner, difficulty accepting infertility-specific gender differences or concerns about the future of the relationship.

A result we find remarkable is that stress resulting from the relationship to their own mother of women with reproductive problems appeared to connect significantly with both depression and trait anxiety. While previous literature demonstrates the stress-relieving effect of perceived social support from the family in general, the attitude of the infertile woman’s mother and its effect on her coping with infertility have not been largely studied. In the few works on this topic, mothers proved to have a more supportive role for infertile women than fathers and siblings [49]; however, mothers’ rejection or ambivalence predicted the depression of less socially skilled women with fertility problems [31]. Our result corroborates that, if support from the mother, an essential element of the family network, is insufficient, especially when the woman herself is struggling with maternal role attainment, this may well increase the distress level of the infertile woman.

Despite the fact that a higher income was more frequent among infertile women than among their fertile counterparts, financial stress was another important entity associated with the trait anxiety of infertile women. This result is somewhat ambiguous because, while increased presence of financial problems put an additional strain on couples struggling with infertility [32], in Hungary up to 5 IVF cycles are covered by mandatory social health insurance plans [50]. However, we hypothesize that financial problems are still significant because infertility entails several additional expenses beyond the cost of IVF treatments (expenditure of medications, travelling to fertility centers and lost work time).

We did not find illness stress to be related to either depression or trait anxiety among our infertile subjects. Since reproductive problems are often dealt with from a medical perspective [1], the question about illness stress was intended to detect emotional distress resulting from a chronic illness, which in the case of infertility often reaches the severity found in sufferers from cancer or heart disease [33]. The fact that illness stress lacked connection to depression and anxiety in our study is possibly due to two factors: 1) that the Fertility Problem Inventory captures infertility stress in a more comprehensive manner and, indeed, two of its subscales were associated with distress in our study, and 2) we measured illness stress with only one question formulated in a much too general way.

One of the strengths of our survey is that we used common, well-validated questionnaires and an analytical design to assess trait anxiety and depression (STAI-T and BDI, respectively), while also measuring infertility-related stress (FPI). Additionally, we investigated a wide array of stress sources as potential psychological background factors behind distress. Our study also has the advantage of including subjects never having received fertility treatment (23.1% of our infertile sample). A novelty of our study stands in examining the effect of social support on psychological well-being, thus being able to point at the crucial importance of maternal support. Finally, our respondents were reached not only personally but also through the internet, thus mounting to a broader sample of women with or without fertility impairments.

However, our study has some shortcomings and it does leave some questions open for future research. First, our study included a relatively small sample, especially for the infertile group with no assisted reproduction, and all subsamples consisted only of women. Second, despite having exact data on present fertility intentions of the fertile group as well, thus being able to rule out secondary infertile women, there is a possibility of including in the fertile group women with children who are in fact secondary infertile, but have no knowledge about this (because of using contraception or trying to conceive for less than a year). Similarly, fertility intention could be a potential source of misclassification of the infertile group: theoretically a woman (despite an active sexual life without contraception, even in the absence of a “willingness to have a baby”) can be biologically infertile. However, we aimed to study “involuntary childlessness”, therefore, we considered “willingness to have a child” a relevant criterion for infertility. Then again, the classification criteria of our sample (infertile vs. fertile) correspond to categorization methods used in previous studies [39–41]. Third, sources of stress were measured with only one question each, formulated in a much too general way.

The generalization of our results is also limited by self-selection in the case of online respondents, whose meeting of the inclusion criteria cannot be determined clearly. We did not verify the identity of online respondents (no phone calls or clinic follow-ups), which may have influenced the results through selection bias. Also, the groups from clinical settings and online were not examined separately, due to the low number of cases from clinical settings. However, recent studies have proved results based on online data to be as authentic as those obtained through clinical data [17, 30]. Further, women with a high level of education are overrepresented in both samples. Therefore, while this rules out the possibility of educational stage being a confounding variable behind our results, it is uncertain whether our findings can be generalized for women of a lower education level. Finally, since our study had a cross-sectional design, it is impossible to draw meaningful conclusions about the routes of causation between correlated variables.

For future studies we suggest backing up the quantitative analyses with qualitative methods including in-depth interviews with a strategically selected sample (e.g. infertile women with their partners and/or other family members such as mothers). We also suggest using a longitudinal design in order to clarify the potentially circular causation between infertility-related distress and impaired reproductive potential.

## Conclusions

Compared to fertile women, infertile females are characterized by a significantly worse psychological status in terms of trait anxiety and depressive symptoms. Among infertile women, age, social and sexual concern, maternal relationship stress and financial stress were significantly related to distress. This study points at the necessity of specific psychological interventions, presently absent from the Hungarian public healthcare routine, for women struggling with infertility, to help them manage potential mental health problems and meet their reproductive goals.

## Abbreviations

ART: Assisted reproductive treatment
IVF: In vitro fertilization
